# The Foreign Journals: Anatomy and Physiology

**Published:** 1839-10

**Authors:** 


### PART THIRD.

### Selections from the British and Foreign Journals.

---

### I. THE FOREIGN JOURNALS.

### ANATOMY AND PHYSIOLOGY.

*On the Physiology of Nutrition.* By J. F. Simon.

The following experiments were undertaken with a view to determine whether the stomach of the child possesses the same properties of coagulating milk as the stomach of the calf.

I. A piece of the stomach of a child, which had died immediately after birth, was introduced into a dish containing cow's milk. Into a second dish, containing an equal quantity of milk, a portion of a calf's stomach was introduced; and a third dish contained milk, without any addition. The three vessels were placed in the water-bath, at a temperature of 30° R. In twenty-eight minutes the milk containing the calf's stomach had coagulated, but that in the other two dishes, after the lapse of an hour, remained uncoagulated These two dishes were left standing during the night in a temperature of 17° R; in the morning the milk containing the portion of child's stomach was still uncoagulated; that in the other dish had coagulated from acidity.

II. A portion of the stomach of a child, five days old, having been previously well washed, was introduced into cow's milk, as in the preceding experiment, with a like result. A quantity of the colostrum of the child's mother was then obtained and divided into three portions of about six drachms each. Into one portion a piece of the child's stomach was introduced, into another a piece of calf's stomach, and to the third no addition was made. The milk of the first and second portions began to coagulate nearly at the same time; but the coagulum of the first was firmer and the whey clearer than those of the other. The third portion did not coagulate. From these experiments it is inferred that the stomach of a mammæ is adapted for the coagulation and digestion of the milk of its own species only.

III. Into each of three test tubes a portion of curd of cow's milk, coagulated by runnet, from which the whey had been expressed, was introduced. The curd, in two of the glasses, was covered by a piece of fresh calf's stomach, and in the third by a piece of child's stomach. A fourth glass contained a portion of the curd of woman's milk, coagulated by the stomach of a child. Into each of the glasses a quantity of acidulated water was now poured, and they were placed in the temperature of 30° R. In an hour and a half artificial digestion commenced in all four glasses; and in nineteen hours the curd of the fourth glass was, with the exception of a few flakes, completely dissolved. In twenty-three and twenty-four hours the first and second glasses had advanced to an equal stage; but at the end of thirty hours, when the experiment was broken off, the curd contained in the third glass was still not fully dissolved. It should be remarked, however, that the quantity of curd in the third glass was only about a third of that contained in each of the others; but the portion of stomach was small in proportion. Experiment showed that the casein is changed by the artificial digestion into albumen. The solution of the curd coagulated on the application of heat, and comported itself towards the chemical reagents precisely as albumen. *Müller's Archiv.* Heft i. 1839.

*On the Production of Urea in the Animal Body.* By Dr. R. Marchand.

The following experiments were undertaken with a view to determine whether urea can be detected in the blood of an animal which has fasted for several days. With a view to lessen the cruelty of the experiment, Dr. Marchand fed the animal upon pure candi-sugar, a substance which contains no nitrogen, and which could therefore exert no influence upon the result of the experiments.

A large and powerful shepherd's dog was fed for fourteen days upon milk, in order to ascertain the quantity of urea contained in the urine of an animal thus simply nourished. During the first five days the quantity was 2·6 per cent.; during the next five days it was 3 per cent., at which figure it remained during the rest of the experiment. The animal was now fed upon pure sugar and distilled water, and it consumed daily about 10 oz. sugar. During the first six days the urine contained 2·8 per cent. urea; in the next five days the quantity was 2·4 per cent.; and in the next five 1·8 per cent. The animal was now very weak and lean, but there were no ulcerations of the cornea. The diet was again changed, and the dog was fed on milk and bouillon; but the increase of the quantity of urea did not correspond with the rapidity with which the animal recovered its flesh. The quantity of urea was only 2·4 per cent. after the dog had recovered its former good condition, and it did not regain its full proportion of 3·2 to 3·35 per cent. for some days afterwards. Sugar and water were again given, and in eight days the proportion of urea was 2 per cent. The abdomen of the animal was now laid open, and a ligature was applied to the renal nerves, an operation which Dr. Marchand regards as equivalent to extirpation of the kidneys. The incisions soon healed, and nothing remarkable occurred for six days, when vomiting ensued, but the matter vomited was too small in quantity to allow of chemical examination. Ten days after the operation the jugular vein was opened, 3 lbs. of blood were taken, and the animal died immediately. The blood yielded 4·88 grammes of nitrate of urea.

Dr. Marchand is of opinion that urea exists in the blood in the healthy state, although in extremely minute quantities. The nicest test for the detection of urea is the crystallization of chloride of sodium in octoedrons, which ensues, when it is present. Dr. M. found that $\frac{1}{10}$ or $\frac{1}{20}$ urea dissolved in 100 to 150 parts of water was sufficient to cause the production of at least some octoedral crystals of the salt; and, as yet, urea is the only substance which we know to possess this property. Twenty pounds of the serum of cow's blood, treated with alcohol, to free it from albumen, and then evaporated to dryness, gave a residue which was exhausted with alcohol and again evaporated. The residue thus obtained, on being dissolved in water and mixed with salt, caused it to crystallize in octoedrons.

*Müller's Archiv.* Heft i. 1839.

---

*Observations on the Plague.* By Dr. A. Bulard.

[This is a paper of some value on the morbid anatomy of the plague, communicated to the Editor by Dr. Bulard, while on a recent visit to Berlin. As this gentleman spent six years in Turkey and Egypt in investigations respecting that disease, his observations are certainly entitled to consideration. We can only afford room for some of the most important particulars, which we the less regret, as we are promised a four-volume work on the subject, by and by. The dissections were made at various intervals, from half an hour to twenty hours after death, and in every instance with uncovered hands, and without using any disinfecting materials.]

*Skin.* There were always more or less livid patches on the anterior surface of the neck and chest in whites, as well as ordinarily on the scrotum and labia pudendi; it is presumable, therefore, that the same existed in people of colour. The petechiæ, observed during life, persisted after death; and the buboes and carbuncles always collapsed.—*Muscular system.* Slight cadaveric rigidity, diminished cohesion of muscular tissue, which is soft, somewhat moist, and light coloured.

—*Nervous system.* Sinuses of dura mater and meningeal vessels generally very full of blood; membranes themselves normal; medullary substance of brain presents numerous minute accumulations of blood on section; the cortical substance has generally a polished appearance; consistence of the organ diminished. Sympathetic nerve neither red nor softened; its ganglia everywhere healthy, petechiæ in some cases in the lower end of its thoracic portion.—*Sympathetic system.* Here existed the only really constant, the most extensive, best-marked, and at the same time least-known morbid changes. The glands varied in size from that of a pistachio-nut to that of a goose-egg, and upwards; in colour from that of the cortical substance of the brain to the deepest livid; in consistence, from that of scirrhus to that of putrid detritus. Considerable effusion of blood is found in the abdomen, on the same side as the inguinal buboes; and knotty tumours extending in the course of the lymphatic vessels up to the diaphragm. The substance of the glands presents the evidence of every stage of morbid change from sub-inflammation to suppuration; the lymphatic vessels appear never to participate in the diseased changes. Effusion of blood takes place in the axilla also, as well as into the integuments of the chest, when the disease is centralized in the glands of those regions. The lymphatic system is not diseased in all its parts at one and the same time; buboes never coexist in both axillæ, both groins, both cervical and popliteal regions, nor are the homonymous glands of both sides ever simultaneously attacked.—*Respiratory organs.* Lungs crepitating; bronchial mucous membrane healthy.—*Heart, &c.* Pericardium frequently contains red serosity; right side of the heart completely filled with clotted black blood, often with fibrinous concretions; similar blood in the veins, which often presents something like drops of oil on its surface; arteries generally empty and healthy. —*Organs of digestion.* Softening of the membranes was an ordinary appearance; the stomach frequently loaded with blackish fluid; its external membrane ordinarily palish yellow, thickened, and softened; internal surface marked with petechiæ, and in advanced cases ulcerated, a state never observed in the small intestine; the vermiform appendix of twice or thrice the natural size; Brunner's and Peyer's glands normal.—*Secretory organs.* Liver loaded with black blood; gall-bladder distended and spotted with petechiæ; its membranes thickened in twelve cases; bile in nothing remarkable. Spleen enlarged to treble or quadruple its normal size, and filled with blood like wine-lees. In four or five cases only was this organ healthy; in six instances it contained carbuncles. Pancreas normal; kidneys usually twice or thrice their natural size, loaded with dark-coloured blood; with an appearance of real hemorrhage in the pelvis.

These lesions are divided by Dr. Bulard into two classes, the primitive and consecutive. The former are those affecting the lymphatic system, and originating in a morbid state of the *lymph* itself. Among the secondary alterations of most importance are those of the *blood,* and they are induced, according to this observer, through the direct communications between the lymphatic vessels and veins; the condition of the blood accounts in its turn for that of the *various organs.* That fluid never presented any buff; its cohesion was found greater than natural during venesection; it emitted a peculiar odour in some cases, and occasionally remained perfectly fluid. It was analyzed thrice, and found to contain in 100 parts the following ingredients:

| | | |
|---|---|---|
| Clot | Water | 35·576 |
| | Fibrine | 00·624 |
| | Colouring matter, with some fibrine, albumen, and fatty matters | 3·800 |
| Serum | Water | 54·420 |
| | Albumen and colouring matter | 4·704 |
| | Extraction | 00·252 |
| | Chloride of potassium and sodium | 00·408 |
| | Carbonate of soda and fatty matters | 00·216 |
| | Distinct traces of sulphurous acid. | |

The black-coloured mass found in the stomach was also analyzed, and 100 parts found to contain:

| | |
|---|---|
| Water . . . . . . | 95·75 |
| Oxide of iron . . . . . | 0·25 |
| Resin . . . . . . | 1·75 |
| Mucus and fat . . . . . | 0·25 |
| Albumen with colouring matter . . | 2·00 |

*Wochenschrift für die gesammte Heilkunde,* No. 42. *Oct.* 1838.

